# Neutrophil migration participates in the side effect of recombinant human tissue plasminogen activator

**DOI:** 10.1111/cns.14825

**Published:** 2024-07-02

**Authors:** Yuyou Huang, Ziping Han, Tong Shen, Yangmin Zheng, Zhenhong Yang, Junfen Fan, Rongliang Wang, Feng Yan, Zhen Tao, Yumin Luo, Ping Liu

**Affiliations:** ^1^ Department of Neurology and Institute of Cerebrovascular Diseases Research Xuanwu Hospital of Capital Medical University Beijing China; ^2^ Beijing Institute for Brain Disorders, Capital Medical University Beijing China

**Keywords:** blood–brain barrier, ischemic stroke, neutrophil, recombinant human tissue plasminogen activator

## Abstract

**Aims:**

Ischemic stroke remains a challenge in medical research because of the limited treatment options. Recombinant human tissue plasminogen activator (rtPA) is the primary treatment for recanalization. However, nearly 50% of the patients experience complications that result in ineffective reperfusion. The precise factors contributing to ineffective reperfusion remain unclear; however, recent studies have suggested that immune cells, notably neutrophils, may influence the outcome of rtPA thrombolysis via mechanisms such as the formation of neutrophil extracellular traps. This study aimed to explore the nonthrombolytic effects of rtPA on neutrophils and highlight their contribution to ineffective reperfusion.

**Methods:**

We evaluated the effects of rtPA treatment on middle cerebral artery occlusion in rats. We also assessed neutrophil infiltration and activation after rtPA treatment in vitro and in vivo in a small cohort of patients with massive cerebral ischemia (MCI).

**Results:**

rtPA increased neutrophil infiltration into the brain microvessels and worsened blood–brain barrier damage during ischemia. It also increased the neutrophil counts of the patients with MCI.

**Conclusion:**

Neutrophils play a crucial role in promoting ischemic injury and blood–brain barrier disruption, making them potential therapeutic targets.

## Graphical abstract

Recombinant human tissue plasminogen activator (rtPA) activates neutrophils, enhancing their migration and adhesion, which participate in the side effect of rtPA.

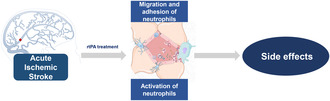

## 1 INTRODUCTION

Ischemic stroke has always been a challenge in medical research due to its high morbidity, disability, mortality, and recurrence rates. While recent studies, including DAWN,^1^ DEFUSE‐3,^2^ and MR CLEAN‐LATE,^3^ have shown advances in endovascular treatment using imaging modalities, effective treatments for ischemic stroke remain limited. Intravenous thrombolysis with recombinant human tissue plasminogen activator (rtPA) is the only effective treatment for vascular recanalization in patients with acute ischemic stroke.^4^ However, patient response to rtPA thrombolysis remains highly variable, even with standardized assessment. Nearly 50% of patients still experience adverse clinical outcomes, such as failure to recanalize and hemorrhagic transformation after recanalization after rtPA exerts its thrombolytic biological effect, indicating the lack of effectiveness of recanalization.^5^ The specific mechanisms underlying ineffective reperfusion remain unclear. However, recent studies have shown that the activation, migration, and infiltration of immune cells, especially neutrophils, may be significant during the early stages of cerebral ischemia. This affects the prognosis of rtPA thrombolysis.^6^

Neutrophils are the first cells to accumulate in cerebral microvessels and venules, appearing 15 min after infarction and peaking at 24 h.^7^ Neutrophils release various inflammatory and chemotactic factors, matrix metalloproteinases, and reactive oxygen species after infiltrating the ischemic tissues. These substances attract more immune cells and exacerbate damage to the blood–brain barrier (BBB) and brain tissue. Studies have shown a correlation between higher neutrophil counts before thrombolysis and worse prognosis.^8^ They exhibit a neurotoxic phenotype and damage neurons when invading the ischemic brain tissue. Neutrophil recruitment exacerbates tissue damage by releasing reactive oxygen species (ROS), proteases, and proinflammatory cytokines. The migration and infiltration of neutrophils into ischemic sites are considered key factors in promoting ischemic injury and BBB damage. Adherent neutrophils are the early factors involved in BBB destruction. RtPA can induce a significant release of neutrophil extracellular traps into brain tissue. This triggers the release of inflammatory factors and aggravates BBB damage and cerebral hemorrhage. Current neutrophil treatments for cerebrovascular diseases mainly focus on inhibiting neutrophil infiltration, the release of neutrophil inflammatory factors, or neutrophil granule function to reduce infarct size and improve neurological outcomes.^9^, ^10^, ^11^

Therefore, the main objective of this study was to determine whether the thrombolytic effects of rtPA are due to its effects on neutrophil migration, adhesion, and activation. We hypothesized that this effect may result in ineffective reperfusion after rtPA treatment. By exploring these mechanisms, new treatment strategies may be developed to improve the safety and effectiveness of rtPA thrombolytic therapy and patient prognosis.

## 2 MATERIALS AND METHODS

### 2.1 Human samples and data collection

Human samples were collected in accordance with the Declaration of Helsinki and were approved by the Research Ethics Committee Review Board of Xuanwu Hospital, Capital Medical University, Beijing, China (Clinical Research [2021] No. 069). All enrolled individuals provided written informed consent for the study involving their blood samples. A cohort of patients with middle cerebral artery (MCA) stroke, known as major cerebral ischemia (MCI), was recruited at the Xuanwu Hospital between November 2021 and November 2022. Eligible patients had an age range of 18 to 80 years and were confirmed to have MCI by head computed tomography (CT) or magnetic resonance imaging (MRI). They also had unilateral MCI affecting at least two‐thirds of the MCA territory within 48 h of stroke onset. The exclusion criteria included a severe bleeding tendency, active bleeding, or hematological diseases; severe infections; malignant tumors; renal or liver failure; major surgery within the past 6 months; or a modified Rankin Scale (mRS) score of more than 2 points before the onset of MCI. Upon admission, patients underwent standard neurological and general medical assessments, and the diagnosis of MCI was confirmed based on established guidelines. Seventeen patients with MCI were randomly assigned to either receive rtPA therapy or not. All patients received comprehensive follow‐up care, with their functional prognosis assessed using the mRS score at 6 months. Blood samples from patients with MCI were collected on the second, fourth, seventh, and fourteenth days post‐onset using EDTA anticoagulant vacuum tubes via venipuncture upon their arrival at the emergency room. Two sets of 4 mL blood samples were collected per patient and immediately centrifuged at 4°C for 10 min at 200 × *g*. The resulting supernatant was preserved as plasma for subsequent biochemical analysis. In addition, white blood cell, lymphocyte, and neutrophil counts were measured both before and after rtPA therapy.

### 2.2 Enzyme‐linked immunosorbent assay

The plasma Myeloperoxidase (MPO) and Neutrophil Elastase (NE) concentrations were measured using a human enzyme‐linked immunosorbent assay (ELISA) kit (Xinbosheng, China). The plasma ICAM‐1 concentrations were measured using an ELISA Kit for human s‐ICAM (orb1670793) (Biorbyt, Wuhan, China). The plasma VCAM‐1 concentrations were measured using a human VCAM‐1 ELISA kit (EHC123.96.10) (NeoBioscience, Shenzhen, China). The test was performed according to the manufacturer's instructions.

### 2.3 RNA Extraction and real‐time quantitative polymerase chain reaction

Total plasma RNA was extracted using the TRIzol Reagent (Cat#15,596,026; Invitrogen). Following nanodrop quantification, 1 μg of total RNA with a volume of 20 μL underwent reverse transcription using the HiScript III 1st Strand cDNA Synthesis Kit (Vazyme, Nanjing, China) for RNA and miRNA First‐Strand cDNA Synthesis (tailing Reaction; Cat#B532451; Sangon Biotech, Shanghai, China) for miRNA, following the manufacturer's instructions. The resulting cDNA was used for real‐time quantitative polymerase chain reaction (RT‐qPCR) with Taq Pro Universal SYBR qPCR Master Mix (Vazyme, Nanjing, China) on a StepOnePlus Real‐Time PCR System (Roche, LightCycler 480 II). Universal U6 and human GAPDH served as controls for miR‐27b and the target mRNA, respectively, in quantifying original RNA concentrations using the 2 − ΔΔCT method. Each qPCR assay was performed in triplicate and independently repeated three times. The primer sequence (5′–3′) was AGCATAATACAGCAGGCACAGAC.

### 2.4 Animals

Adult male Sprague–Dawley rats weighing 300–320 g (SPF (Beijing) Biotechnology Co., Ltd.) were used in this study. The experimental procedures were approved by the Institutional Animal Investigation Committee of Xuanwu Hospital, Capital Medical University, and they adhered to the Care and Use of Laboratory Animals guidelines of the National Institutes of Health and ARRIVE guidelines. A total of 64 rats were randomly assigned to four groups: Sham, middle cerebral arterial occlusion (MCAO) (MCAO with 1 mL saline injection, IV as soon as reperfusion), and rtPA (MCAO followed by 1 mL rtPA infusion and 9 mg/kg IV as soon as there was reperfusion). All investigators conducting behavioral testing, histological analyses, and other assessments were blinded to the treatment allocation.

### 2.5 MCAO Model

A suture model of transient cerebral artery occlusion in rats was used to simulate mechanical thrombectomy after ischemic stroke. Anesthesia was induced with 5% isoflurane and maintained with 1%–2% isoflurane, 70% N_2_O, and 30% O_2_ through a facemask. The right middle cerebral artery was occluded for 90 min, and this was followed by reperfusion. The MCAO model was established using the intraluminal suture occlusion method, as previously outlined. Rats that did not display signs of ischemic injury based on the Longa score or those that died before the conclusion of the study were excluded from further analyses.

### 2.6 Neurological deficits

After surgery, neurological deficits were assessed using the Zea‐Longa 5‐point and modified neurological severity score scales 24 and 72 h post‐reperfusion. Tests were conducted by blinded observers.

### 2.7 Infarct volume and histological analysis

The cerebral infarct volume was evaluated 72 h after MCAO. Rats anesthetized with 1% pentobarbital sodium were sacrificed, and their brains were coronally sliced into 2‐mm sections on ice. Brain slices were incubated with 2% 2,3,5‐triphenyl tetrazolium chloride (TTC; T8877; Sigma‐Aldrich, USA) to determine the infarction volume. ImageJ software was used to analyze the brain images. To account for swelling, the relative infarct territory was determined by subtracting the noninfarcted area in the ipsilateral hemisphere from that in the contralateral hemisphere. The infarct volume was calculated by integrating the areas of infarction in the second and fourth hemispheres. Hematoxylin–eosin (HE) staining, analyzed using ImageJ, revealed tissue loss in ischemic brain tissues.

### 2.8 Immunofluorescence

For immunofluorescence (IF), brain tissues obtained 72 h post‐MCAO were fixed in 4% paraformaldehyde for >48 h. Paraffin coronal brain slices with a thickness of 4 μm were incubated with a primary antibody against lymphocyte antigen 6 complex, locus G (Ly6G) (53,515, Santa Cruz Biotechnology, USA), followed by donkey anti‐mouse Alexa Fluor‐conjugated secondary antibodies (1:250, Jackson Immuno Research). All slices were counterstained with 4′,6‐diamidino‐2‐phenylindole dihydrochloride (DAPI; Cat#0100–20; Southern Biotech, USA). Double immunofluorescence labeling for the lymphocyte antigen 6 complex, locus G6D (Ly6G), hepatocyte growth factor (HGF) (26881‐1‐AP, Proteintech, USA), and cellular‐mesenchymal epithelial transition factor (c‐Met) (25869‐1‐AP, Proteintech) was performed, followed by incubation with the secondary antibody and DAPI. Histological images were acquired using a Nikon 80i microscope (Tokyo, Japan) with standardized photographic settings.

Cerebral microvessels were isolated from the brain after 90 min of ischemia and 72 h of reperfusion and smeared for immunofluorescence staining. Double immunofluorescence labeling for Piezo1 (15939‐1‐AP, Proteintech, USA) and neutrophil cytosolic factor 4 (NCF4) (14648‐1‐AP, Proteintech, USA) was performed, followed by incubation with the secondary antibody and DAPI.

### 2.9 Co‐culture system of human brain microvascular endothelial cells and human neutrophil‐like HL‐60 Cells

Human Brain Microvascular Endothelial Cells (HBMECs) and human neutrophil‐like HL‐60 cells (ATCC, Rockville, MD, USA) were co‐cultured in a transwell chamber. The cells were used for experiments after fewer than ten passages. They were placed in the upper chamber of the transwell, and the HL‐60 cells were cultured in the lower chamber. Hypoxia was induced in the HBMECs by incubation in a hypoxic chamber for 2 h at an oxygen concentration of 1% in the presence of glucose. The cells were divided into oxygen and glucose deprivation (OGD) plus rtPA (OGD + rtPA) and OGD groups. N‐formylmethionyl‐leucyl‐phenylalanine (fMLP) and 200 μL rtPA (0.1 mg/mL/PBS) were placed in the upper chamber for 4 h to induce cell chemotaxis. Subsequently, the HBMECs were collected to detect infiltration of HL‐60 cells and damage to tight junctions.

### 2.10 Western blotting

Cerebral microvessels were isolated from the brain after 90 min of ischemia and 72 h after reperfusion. Homogenates (20 μg protein) of the microvessels were prepared for western blotting. The HBMECs were prepared for western blot analysis. The primary antibodies used were Ly6G (1:500, Novus Biologicals), matrix metalloprotein‐9 (MMP‐9) (1:500, Santa Cruz Biotechnology), MMP‐2 (1:500, Santa Cruz Biotechnology), and β‐actin (1:2000, Abcam Biotechnology). The protein concentrations are expressed as a ratio to those of β‐actin.

### 2.11 Statistical analysis

The statistical analyses were performed using IBM SPSS Statistics 22 software (IBM Corp., Armonk, NY, USA). The normality of the data was evaluated using the Shapiro–Wilk test. Data with normal distributions were compared using Student's *t*‐test and one‐way ANOVA. They are expressed as mean ± SD. The Mann–Whitney U test was used to compare the data with nonnormal distributions, which are expressed as median (interquartile range, IQR).

## 3 RESULTS

### 3.1 rtPA treatment may activate the neutrophils and increase their infiltration in patients with MCI

**FIGURE 1:**
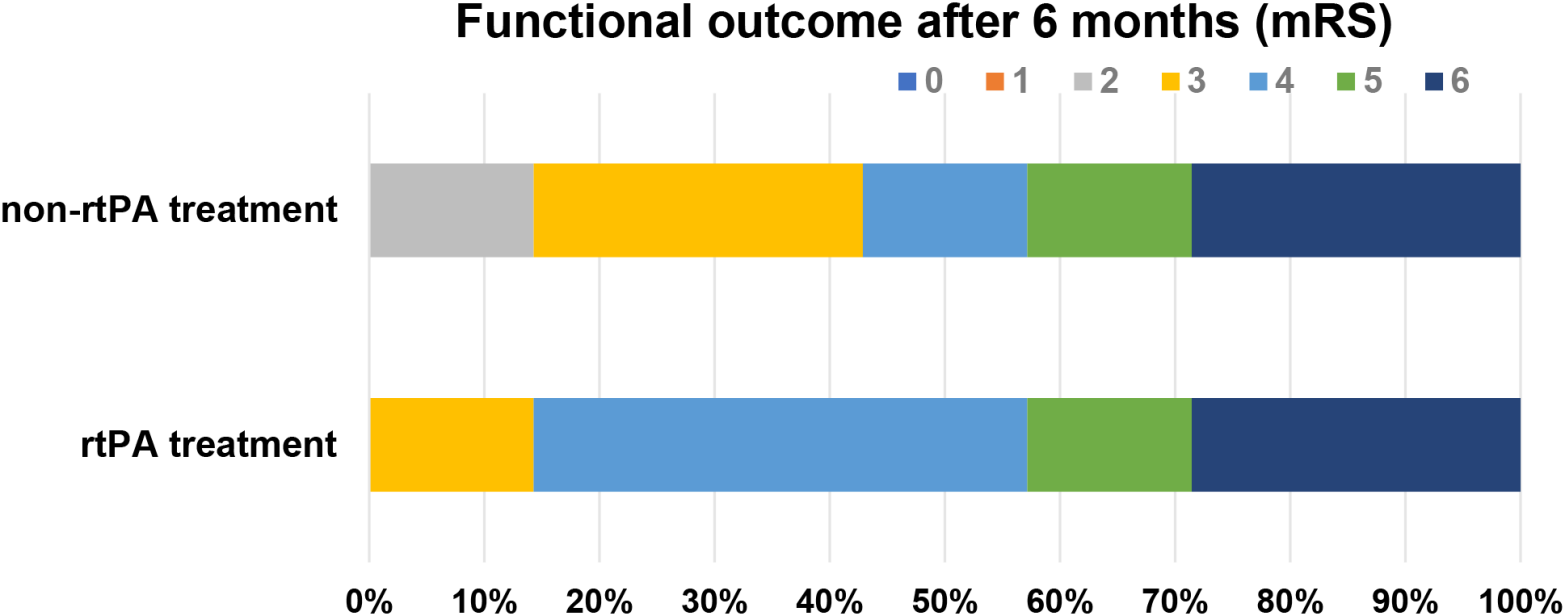
The functional outcome of patients with MCI with and without rtPA therapy. Modified Rankin Score (mRS) of patients with MCI 6 months after stroke.

Fourteen patients with MCI were enrolled in the present study. Each of the rtPA treatment and nonrtPA treatment groups comprised seven participants. Table 1 shows the baseline patient characteristics. There were no notable differences in the clinical baseline characteristics and stroke risk factors, including hypertension, diabetes, hyperlipidemia, coronary heart disease, and atrial fibrillation, of the two groups (*p* > 0.05) (Table 1). We observed no significant difference in the distribution of mRS scores between the rtPA and the nonrtPA treatment groups after 6 months of follow‐up (Figure 1), implying that rtPA treatment did not significantly improve the prognosis of patients, as expected.

**TABLE 1:** Baseline characteristics between MCI patients with and without rtPA treatment.

Parameters	rtPA treatment (*n* = 7)	NonrtPA treatment (*n* = 7)	*p* Value
Demographics			
Age	55.33 ± 10.250	64.38 ± 10.836	0.140
Sex (male)	5	3	0.127
Risk factors			
Hypertension	4	4	1
Diabetes	1	4	0.022
Hyperlipidaemia	1	3	0.127
Coronary artery disease	0	2	0.094
Atrial fibrillation	1	1	1
Heart failure	0	1	0.280
Previous stroke	0	3	0.022
Infection	1	2	0.403
Family history of stroke	5	5	1
Smoking	5	2	0.012
Drinking	3	2	0.403
Clinical features			
Hemisphere(dominant)	3	4	1.000
NIHSS score on admission	21.00 ± 8.343	20.38 ± 11.19	0.431
Median	19	15.5	
Range	12–32	11–40	

**FIGURE 2:**
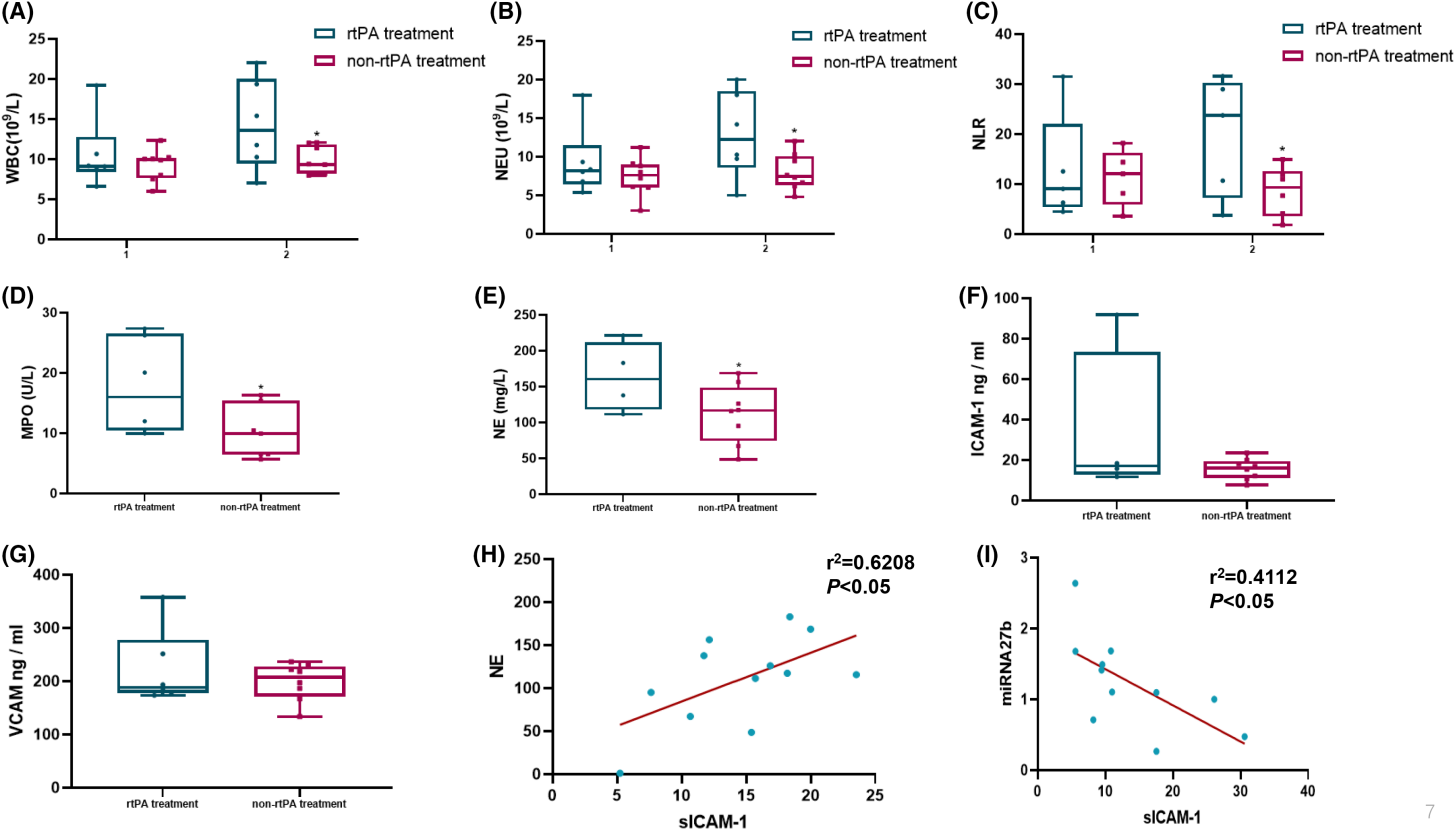
The effect of rtPA treatment on neutrophil activation and infiltration in patients with MCI. (A) White blood cell counts (WBCs) of the rtPA (*n* = 7) and nonrtPA (*n* = 7) treatment groups before and after rtPA therapy. (B) Neutrophil counts of the rtPA and nonrtPA treatment groups before and after rtPA therapy. (C) Neutrophil lymphocyte ratios (NLRs) of the rtPA and nonrtPA treatment groups before and after rtPA therapy. (D) Plasma myeloperoxidase concentrations of the rtPA and nonrtPA treatment groups after rtPA therapy. (E) Plasma neutrophil elastase concentrations of the rtPA and nonrtPA treatments after rtPA therapy. (F) Plasma intercellular adhesion molecule‐1 concentrations of the rtPA and nonrtPA treatment groups after rtPA therapy. (G) Plasma vascular cell adhesion molecule concentrations of the rtPA and nonrtPA treatment groups after rtPA therapy. (H) There was a negative correlation between the plasma sICAM‐1 concentration of the patients with MCI on the fourth day and NE protein expression on the seventh day (*r* ^2^ = 0.3356, *p* < 0.05). (I) There was a negative correlation between the plasma sICAM‐1 concentration of the patients with MCI on the seventh day and the miRNA27b concentration on the fourteenth day (*r* ^2^ = 0.4112, *p* < 0.05). Results are shown as mean ± SD (*p < 0.05; Student *t*‐test).

Considering the change in the neutrophil‐to‐lymphocyte ratio (NLR) as an independent prognostic factor for various diseases, we analyzed the impact of rtPA treatment on NLR. Routine blood test results indicated no significant differences in the white blood cell count, neutrophil count, or NLR values of the rtPA and nonrtPA treatment groups before rtPA treatment (*p* > 0.05, Figure 2A–C). However, on the second post‐treatment day, the patients receiving rtPA treatment had significantly higher white blood cell counts, neutrophil counts, and NLR values than those who were not (*p* < 0.05, Figure 2A–C).

These results imply that rtPA treatment may activate neutrophils, and this contributes to its diverse outcomes. We determined the concentrations of NE and MPO in the plasma of patients with MCI, along with the expressions of ICAM‐1 and VCAM. The expressions of ICAM‐1 and VCAM are known to facilitate neutrophil migration from blood to affected tissues. For the patients with MCI, rtPA treatment yielded significantly higher plasma concentrations of MPO and NE, which were associated with neutrophil degranulation, than the nonrtPA treatment group (*p* < 0.05, Figure 2D,E). The concentrations of ICAM‐1 and VCAM, which are responsible for inducing neutrophil migration, increased after rtPA treatment, but the differences were not statistically significant (*p* > 0.05, Figure 2F,G).

Using linear regression analysis, we identified positive correlations between the plasma ICAM‐1 protein concentrations on the second day after MCI onset and plasma NE protein expression on the seventh day (Figure 2H, *r* ^2^ = 0.6208, *p* < 0.05). In addition, negative correlations were observed between ICAM‐1 expression on the fourth day of onset and miRNA27b expression on the fourteenth day in the plasma (Figure 2I, *r* ^2^ = 0.4112, *p* < 0.05). These findings suggest that elevated plasma ICAM‐1 concentrations in patients with MCI may activate neutrophils, which is associated with reduced miRNA27b expression. This relationship may contribute to vascular endothelial damage and diminished vessel regeneration, potentially leading to an unfavorable prognosis for patients with MCI.

### 3.2 Effects of rtPA treatment on rats with MCAO

**FIGURE 3:**
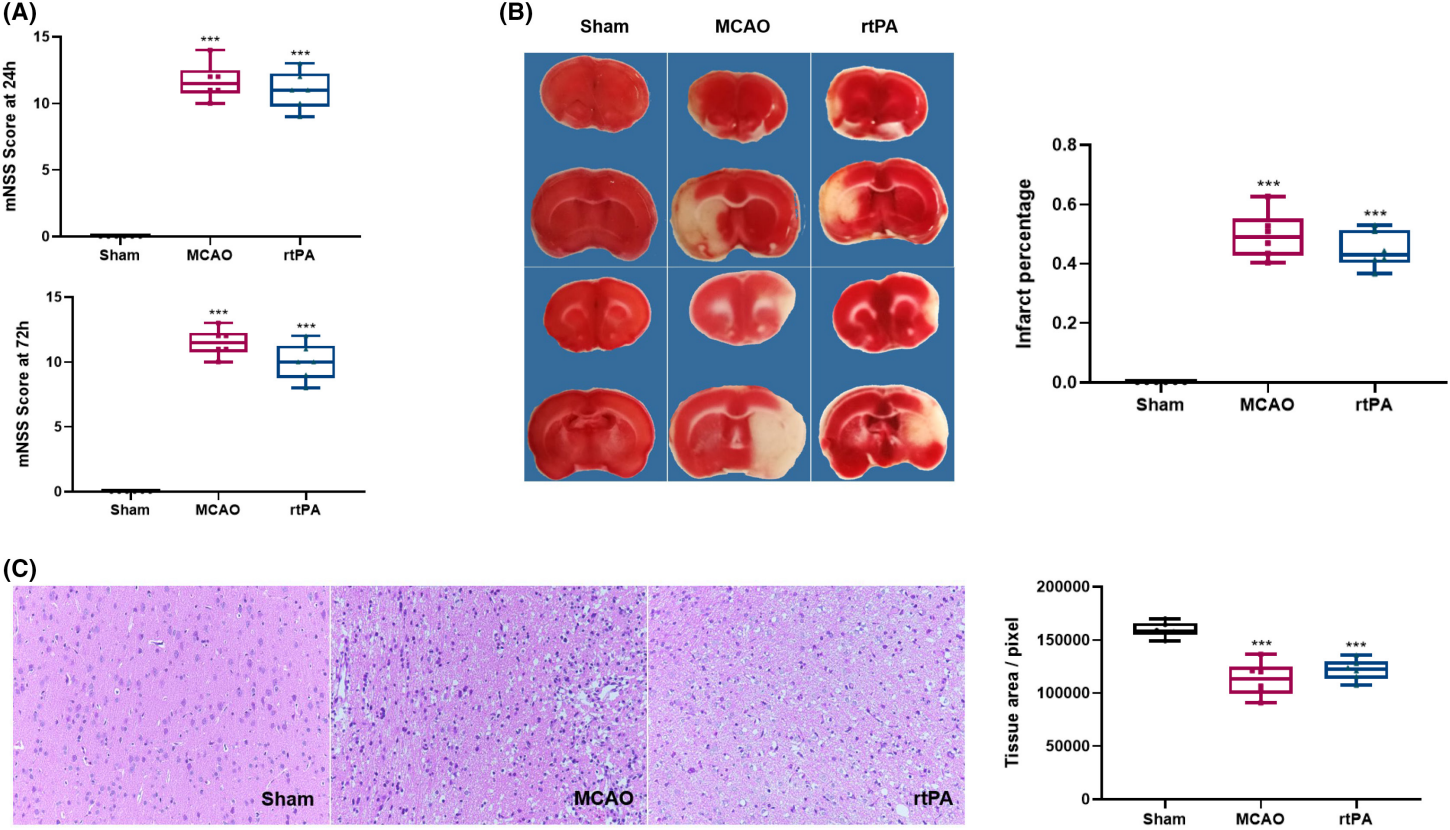
The effect of rtPA treatment in MCAO rats. (A). The neurological function scores of the rtPA and MCAO groups showed no statistical difference after 24 and 72 h of reperfusion (*p* > 0.05). Results are shown as mean ± SD, *n* = 6, (one‐way ANOVA). (B) TTC staining showed that the infarct volume of the MCAO rats after 72 h of reperfusion was not significantly different for the rtPA and MCAO groups (*p* > 0.05). Results are shown as mean ± SD, *n* = 6 (one‐way ANOVA). (C) HE staining showed that the arrangement of brain tissue in the rtPA group was irregular relative to that in the Sham group, and the tissue area was significantly reduced. Compared with the MCAO group, the brain tissue damage in the rtPA group was milder, but significant interstitial edema was observed. Compared with the Sham group, the brain tissue slice areas in the rats in the MCAO and rtPA groups were significantly reduced (***p < 0.001). Results are shown as mean ± SD, *n* = 6 (one‐way ANOVA).

The effect of rtPA treatment on cerebral ischemia–reperfusion injury in rats was evaluated using the modified neurological severity score at 24 and 72 h after cerebral ischemia–reperfusion. Proprioception was observed in three groups of rats (sham, MCAO, and MCAO+rtPA groups) after 24 and 72 h of ischemia–reperfusion. At 24 h after reperfusion, there was almost no difference between the neurological function scores of the rtPA and MCAO groups. After 72 h of reperfusion, the neurological function score of the rtPA group was lower than that of the MCAO group, but the difference was not statistically significant (Figure 3A). After 72 h of reperfusion, there was no significant difference between the cerebral infarct volumes of the rtPA and MCAO groups (Figure 3B). Assessment of the rat brain tissue sections observed using HE staining showed that the brain structure of the rats in the Sham group was clear and complete, and the cells were ordered. The neurons had an abundant cytoplasm, were lightly stained, and had centrally located nucleoli. The density of the intercellular spaces was high. There were no signs of edema or infiltration of inflammatory cells. In contrast, the MCAO group showed irregularly arranged cells and a reduced cell count. The apoptotic cells in the MCAO group had condensed nuclei, dense chromatin, and dark staining. The interstitial fluid was loose and edematous, accompanied by inflammatory cell infiltration. The brain tissue damage was lesser in the rtPA group than in the MCAO group, but there was still significant interstitial fluid edema. Furthermore, there was no significant difference in the brain tissue area relative to that in the MCAO group (Figure 3C).

### 3.3 rtPA increased the infiltration of neutrophils and upregulated the expression of HGF and c‐Met in the infiltrated neutrophils

**FIGURE 4:**
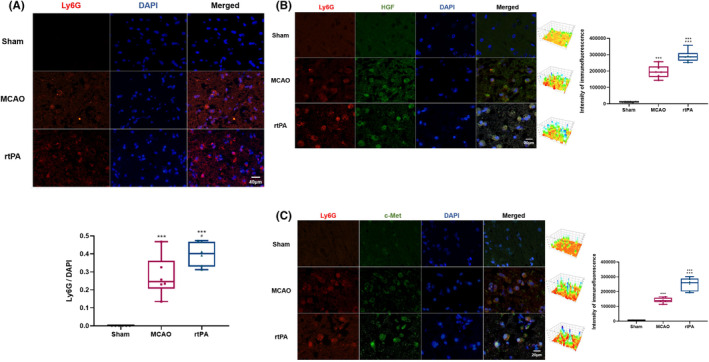
rtPA increases the infiltration of neutrophils and upregulates the expressions of HGF and c‐Met in the infiltrated neutrophils. (A) Neutrophil infiltration in brain tissue at 72 h after reperfusion is detected by immunofluorescence labeling labeling of Ly6G in paraffin sections of brain tissue of rats with MCAO after 72 h of reperfusion. The blue channel represents nuclear DAPI staining. (B) Neutrophil infiltration in brain tissue at 72 h after reperfusion is detected by double immunofluorescence labeling labeling of Ly6G and HGF in paraffin sections of brain tissue of rats with MCAO after 72 h of reperfusion. The blue channel represents nuclear DAPI staining. (C) Neutrophil infiltration in brain tissue at 72 h after reperfusion is detected by double immunofluorescence labeling labeling of Ly6G and c‐Met in paraffin sections of brain tissue of rats with MCAO after 72 h of reperfusion. The blue channel represents nuclear DAPI staining. ***p < 0.001 vs sham group, #*p* < 0.05, ###*p* < 0.001 vs MCAO group. Results are shown as mean ± SD, *n* = 6 (one‐way ANOVA).

As shown in Figure 4A, the number of Ly6G^+^ cells in the penumbra of MCAO rats was significantly higher than that in the sham group, and the number of Ly6G^+^ cells was further increased in the rtPA group relative to that in the MCAO group. Next, we performed double immunofluorescence staining to evaluate the colocalization of HGF and c‐Met with Ly6G^+^ cells and the expression of HGF and c‐Met in rat brain tissues. We observed that HGF^+^ and c‐Met^+^ cells coexisted with Ly6G^+^ cells in the brain tissues of MCAO rats (Figure 4B,C). Compared with the sham group, the fluorescence intensities of HGF and c‐Met in the Ly6G^+^ cells in the brain tissues of the MCAO group increased significantly (Figure 4B). Compared with the MCAO group, the rtPA group showed further increments of the fluorescence intensities of HGF and c‐Met in the Ly6G^+^ cells (Figure 4C).

**FIGURE 5:**
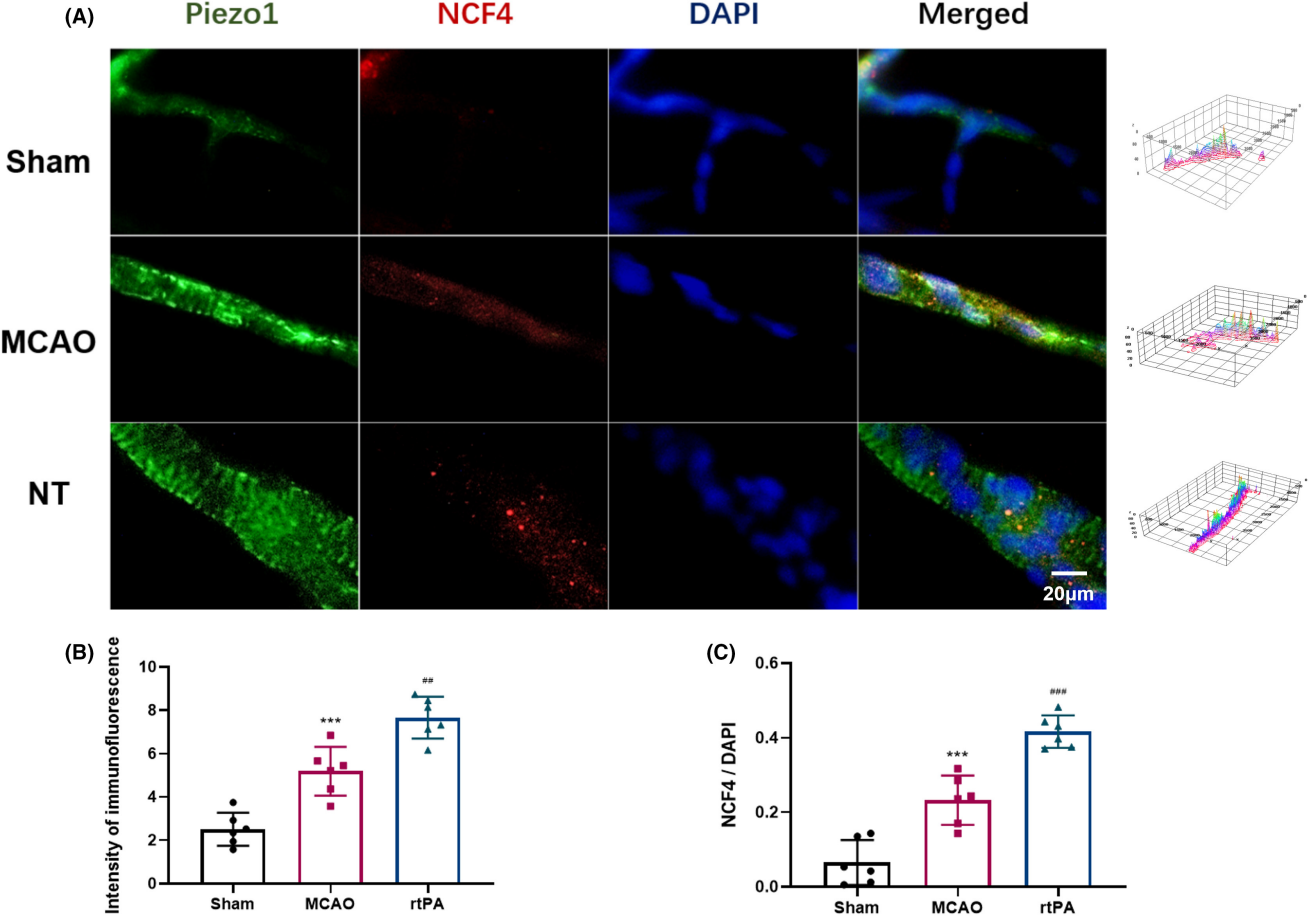
RtPA upregulates Piezo1 expression in the cerebral microvasculature of the rats with MCAO. (A) Double labeling immunofluorescence results of Piezo1 and NCF4 in the cerebral microvasculature of rats with MCAO after 72 h of reperfusion were obtained. The green channel represents Piezo1 immunofluorescence staining, the red channel represents NCF4 immunofluorescence staining, and the blue channel represents DAPI staining of the cell nucleus. (B) The intensity of immunofluorenscence of Piezo1. (C) Quantification of NCF4 positive cells. ***p < 0.001 vs Sham group, ##*p* < 0.001 vs MCAO group, ###*p* < 0.001 vs MCAO group. Results are shown as mean ± SD, *n* = 6 (one‐way ANOVA). (A) Double labeling immunofluorescence results of Piezo1 and NCF4 in the cerebral microvasculature of rats with MCAO after 72 h of reperfusion were obtained. The green channel represents Piezo1 immunofluorescence staining, the red channel represents NCF4 immunofluorescence staining, and the blue channel represents DAPI staining of the cell nucleus. (B) The intensity of immunofluorenscence of Piezo1. (C) Quantification of NCF4 positive cells.

Immunofluorescence staining revealed higher Piezo1 expression in the cerebral microvasculature of the rats in the MCAO and rtPA groups than that in the Sham group after 72 h of reperfusion. In addition, Piezo1 was colocalized with NCF4^+^ cells. Compared with the MCAO group, the rtPA group demonstrated significantly increased Piezo protein expression and a higher number of NCF4^+^ cells (Figure 5).

### 3.4 rtPA increases neutrophil infiltration in brain microvessels and worsens BBB damage in vivo

**FIGURE 6:**
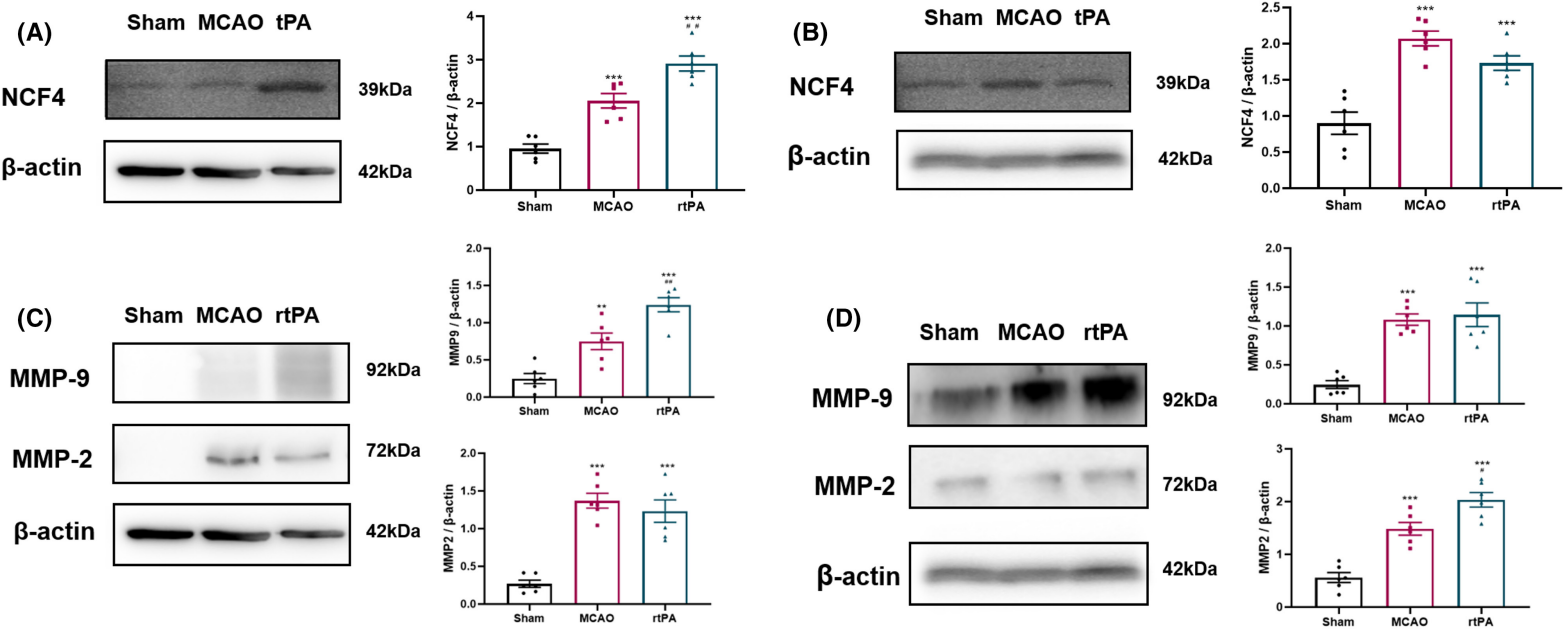
rtPA increases neutrophil infiltration in brain microvessels and worsens blood–brain barrier damage in vivo. (A) Western blot detection of NCF4 in brain microvessels of rats after 24‐h reperfusion. (B) Western blot detection of NCF4 in brain microvessels of rats after 72‐h reperfusion (C). Western blot detection of MMP‐9 and MMP‐2 expressions in brain microvessels of rats after 24‐h reperfusion. (D) Western blot detection of MMP‐9 and MMP‐2 in brain microvessels of rats after 72‐h reperfusion. Results are shown as mean ± SD, **p < 0.01, ***p < 0.001 vs Sham group, #*p* < 0.05, ##*p* < 0.01 vs MCAO group, *n* = 6 (one‐way ANOVA).

Brain microvessels were extracted and lysed to extract the proteins. The expression of NCF4 in the microvessels was analyzed using western blotting. Microvessel infiltration by neutrophils was detected. The results showed that the expression of NCF4 in the microvessels of the MCAO group increased after 24 h of reperfusion relative to that of the sham group, but the difference was not statistically significant. However, the rtPA group showed a significant increase in NCF4 expression relative to that of the MCAO group (Figure 6A). At 72 h after reperfusion, the expressions of NCF4 in the microvessels of the MCAO and rtPA groups were significantly higher than that of the sham group; the expressions in the MCAO and rtPA groups were not different (Figure 6B). Next, we detected MMP expression in brain microvascular samples. The results showed that the expressions of MMP‐9 and MMP‐2 significantly increased in the MCAO and rtPA groups relative to the sham group after 24 h of reperfusion. In the rtPA group, the expressions of MMP‐9 and MMP‐2 further increased; however, the difference was not significant (Figure 6C). After 72 h of reperfusion, the MMP‐9 and MMP‐2 expressions continued to increase in the MCAO and rtPA groups relative to those in the sham group. In addition, the expressions of MMP‐9 and MMP‐2 were significantly higher for the rtPA group than for the MCAO group (Figure 6D).

### 3.5 rtPA increases neutrophil infiltration and worsens BBB damage in vitro

**FIGURE 7:**
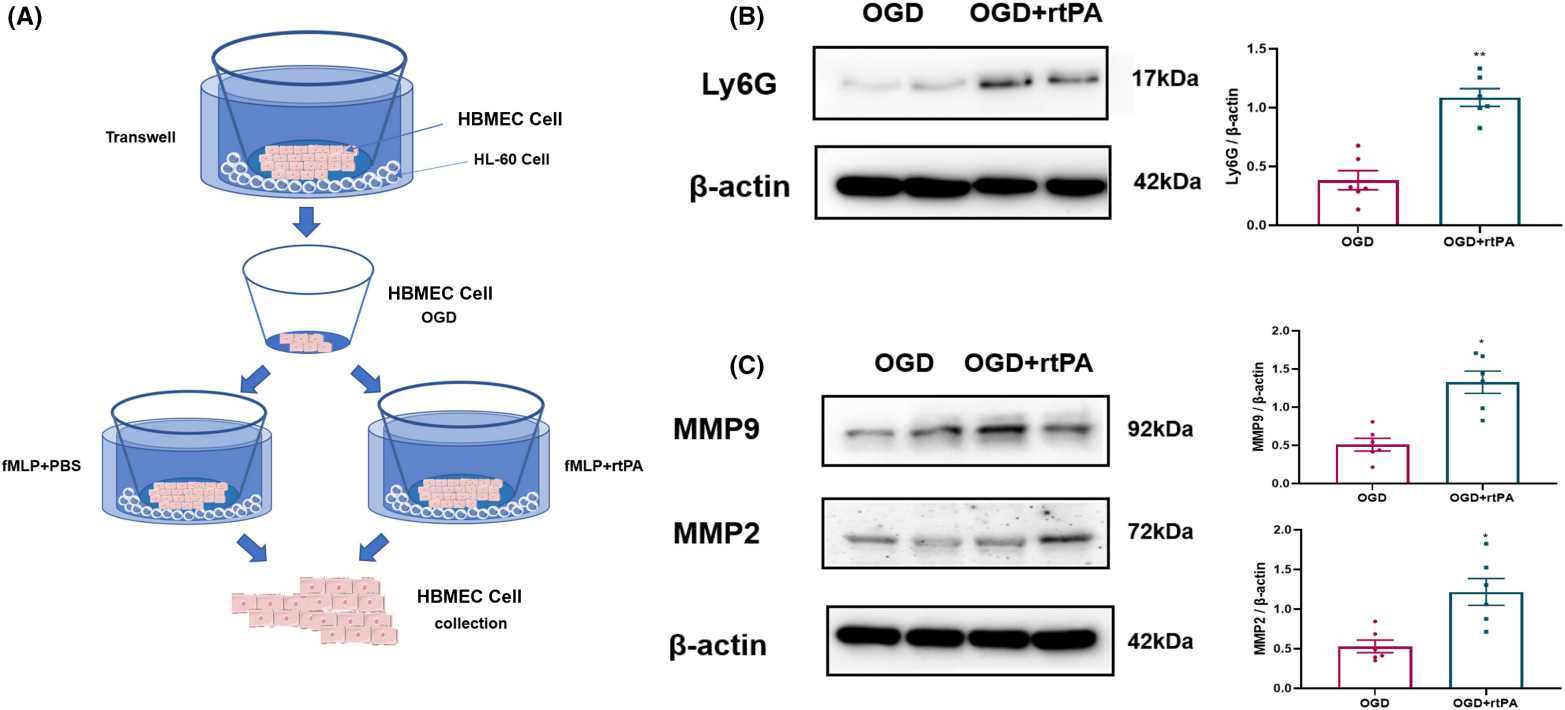
rtPA increases neutrophil infiltration and worsens blood–brain barrier damage in vitro. (A) Human Brain Microvascular Endothelial Cells (HBMECs) and Human Leukemia Cells (HL60) were co‐cultured in a transwell chamber. (B) Western blot detection of Ly6G in HBMECs (C). Western blot detection of MMP‐9 and MMP‐2 expressions in HBMECs. Results are shown as mean ± SD, *p < 0.05, **p < 0.01 vs OGD group, *n* = 6 (Student *t*‐test).

Next, we investigated the effects of rtPA on neutrophil migration and adhesion using Transwell chambers. HBMECs and HL‐60 cells were co‐cultured in Transwell chambers and divided into OGD and OGD + rtPA groups. The neutrophils showed directional migration toward OGD‐treated endothelial cells after exposure to the chemokine fMLP (Figure 7A). Next, we collected the endothelial cells, detected the infiltration of HBMECs by HL‐60 cells, and determined the expressions of MMP‐2 and MMP‐9 in HBMECs. Compared to the OGD group, the OGD + rtPA group showed a significant upregulation of Ly6G in the HBMECs (Figure 7B). In addition, the expressions of MMP‐2 and MMP‐9 in endothelial cells in the OGD + rtPA group were further increased relative to those in the OGD group (Figure 7C).

## 4 DISCUSSION

Drugs targeting the fibrinolytic system remain preferred for the treatment of AIS. Dissolving thrombi and restoring cerebral blood flow remain the most important approaches to salvaging ischemic brain tissues.^12^ As the primary thrombolytic agent used clinically, rtPA is rapidly metabolized. It has a half‐life of 5 min in human plasma. Consequently, adverse effects related to the fibrinolytic activity of rtPA may occur shortly after the infusion. However, adverse clinical events following rtPA treatment mostly occur within 24 h, with a median onset time of 5–10 h. Therefore, these adverse events were likely associated with the nonthrombolytic effects of rtPA. This is consistent with the timing of neutrophil infiltration into the central nervous system, with neutrophils anchoring and rolling on endothelial cells mediated by VCAM and ICAM and traversing tight junctions between endothelial cells via recombinant cytoskeletal structures and intercellular connections.^13^, ^14^

Through in vivo and in vitro investigations, this study demonstrated that rtPA treatment did not improve neurological function or brain injury caused by I/R, as expected. However, rtPA exacerbates the migration and infiltration of neutrophils, intensifying the disruption of vascular tight junctions. Subsequently, we confirmed that rtPA treatment may activate neutrophils in a small cohort of patients with MCI, leading to the upregulation of cell migration mediators. This may be one of the reasons why rtPA treatment failed to effectively improve neurological function in the patients.

Neutrophil activation, migration, and infiltration may contribute to reperfusion failure, thereby affecting rtPA thrombolytic therapy. Moreover, rtPA can directly or indirectly induce neutrophil activation through various pathways, thereby mediating reperfusion injury.^15^ Erdener et al. observed sustained cerebral capillary flow arrest in the salvageable penumbra after reperfusion in mice with MCAO using high‐resolution imaging of capillary circulation. Treatment with anti‐Ly6G antibody effectively mitigated this phenomenon.^16^ Clinical studies have revealed that elevated neutrophil counts are associated with bleeding transformation and poor functional prognosis in patients with AIS after vascular recanalization therapy.^17^ A study published in Nature Communications further corroborated that rtPA can activate neutrophil extracellular traps via pathways such as cyclic GMP‐AMP‐Sting. This exacerbates blood–brain barrier disruption and increases the risk of bleeding transformation. Conversely, neutrophil depletion has been shown to alleviate BBB damage and promote vascular regeneration.^18^ In our investigation of patients with early MCI, we found that NE and MPO released due to neutrophil activation may serve as independent predictors of brain herniation after reperfusion. In addition, the levels of expression of NE and MPO were positively correlated with the neurological function score of patients with large‐area AIS.^19^

Compared with the MCAO group, where neutrophils extensively infiltrated the cerebral microvasculature at 72 h post‐reperfusion, rtPA injection may advance the infiltration of neutrophils as early as 24 h and further mediate brain injury during the early stages of reperfusion.^7^ Neutrophil migration is associated with the disruption of the endothelium.^20^, ^21^ However, recent research suggests that neutrophils can traverse tight junctions without serum protein extravasation, leading to paracellular pathway disruption.^14^ Activated neutrophils release ROS, proteolytic enzymes, and cytokines that affect the cytoskeleton, junctional proteins, and endothelial glycocalyx, thereby impairing tight junctions. In addition, neutrophils enhance protein exchange through endothelial cells by releasing proteases such as MMPs, disrupting junctional complexes, and inducing endothelial cell retraction.^21^ The inhibition of neutrophil interactions by deposited platelets limits vascular injury and reduces tissue damage.^20^

HGF has potent mitogenic effects. As a multifunctional cytokine derived from stromal cells, HGF induces the proliferation of epithelial and fibroblasts and promotes angiogenesis. Experiments have revealed that HGF expression in vascular endothelial and smooth muscle cells stimulates endothelial cell growth through autocrine and paracrine mechanisms.^22^ Studies have indicated that ischemia increases HGF secretion and upregulates HGF receptors in the ischemic tissues.^23^ In our previous study, we identified a significant elevation of HGF concentrations in the plasma of patients with ischemic stroke, suggesting its potential as an independent predictor of adverse stroke prognosis.^24^ c‐MET is a classic tyrosine kinase receptor involved in various physiological and pathological processes. Abundant research supports c‐MET as the HGF receptor on the surface of neutrophils, and activation of this pathway promotes neutrophil proliferation and migration.^25^

The elevation of NLR in the blood of patients with MCI indicates neutrophil activation. Neutrophils play a complex role in the primary immune response during ischemic stroke. Evidence from human and animal models suggests that neutrophils can trigger pathological inflammation in various scenarios.^26^, ^27^ The tendency of neutrophils to damage the surrounding tissues is closely correlated with their activation. Upon migrating to damaged tissues, neutrophils typically clear inflammation through phagocytosis, degranulation, and the formation of neutrophil extracellular traps.^15^ Neutrophil degranulation is associated with adverse outcomes in patients with MCI. MPO and NE are enzymes produced by neutrophils, stored in alkaline granules, and released upon activation, and they lead to inflammation. MPO release may induce oxidative stress, whereas NE can damage endothelial cells and the BBB.^19^, ^28^ In patients with MCI, the plasma concentrations of MPO and NE increased significantly after rtPA treatment. The increased expression of NE may mediate the migration and adhesion of neutrophils, subsequently downregulating the expression of miRNA27b, an miRNA known to induce vascular regeneration.^29^

This study revealed that thrombolytic rtPA treatment activated neutrophils and enhanced their migration and adhesion. This phenomenon may be a potential molecular mechanism underlying the ineffectiveness of recanalization after rtPA treatment. We anticipate that this discovery will pave the way for new research avenues to enhance the safety and effectiveness of rtPA thrombolysis and improve patient outcomes.

## CONFLICT OF INTEREST STATEMENT

Yumin Luo serves as an Editorial Board member of CNS Neuroscience and Therapeutics and is also a co‐author of this article. To minimize bias, they were excluded from all editorial decision‐making related to the acceptance of this article for publication.

## Data Availability

The data that support the findings of this study are available from the corresponding author upon reasonable request.
